# Coupled numerical simulation and strategy optimization of land subsidence caused by groundwater extraction in Langfang City

**DOI:** 10.1371/journal.pone.0348573

**Published:** 2026-05-07

**Authors:** Yongjie Xie, Ge Fu, Haipeng Guo, Jianfeng Qi, Kaijie Guo, Yunlong Wang, Shixiong Tian

**Affiliations:** 1 Hebei International Underground Space Associated Research Centers, Shijiazhuang, China; 2 Hebei Cangzhou Groundwater and Land Subsidence National Observation and Research Station, Cangzhou, China; 3 China Institute of Geo-Environment Monitoring, Beijing, China; 4 Hebei Branch of China National Geological Exploration Center of Building Materials Industry, Baoding, China; Henan Polytechnic University, CHINA

## Abstract

Numerical simulation is an effective method for studying land subsidence caused by groundwater extraction. In this paper, the land subsidence in Langfang City was analyzed by using a coupled fluid-solid three-dimensional numerical simulation. Firstly, four aquifer groups were divided into several aquifers and aquitards, and a finite element model was established. Previous studies often used the Mohr-Coulomb model or linear elastic models to describe the mechanical properties of aquitards, but these models are unable to capture yield characteristics under hydrostatic pressure, whereas the Cam-clay model can reflect this behavior. Based on groundwater level and monitoring data by layered settlement gauges from Danfeng Park, the model was validated. The results indicate that the Cam-clay model effectively simulates the deformation behavior of aquitards. Secondly, 15 different extraction scenarios were used to analyze the variations in pore water pressure in the aquifers, deformation of the aquitards, deformation of 4 aquifer groups, and land subsidence. The study showed that the deformation of aquitards within the pumping-affected zone is predominantly plastic, and it was found that the forms of plastic deformation include strain hardening, strain softening, and the critical state. Further analysis revealed that, under the same extraction volume, deep groundwater extraction had a greater impact on pore water pressure, plastic deformation of the aquitards, and land subsidence compared to shallow groundwater extraction. Finally, a comparative analysis of groundwater extraction scenarios was conducted, and a reasonable extraction scenario was proposed, providing a scientific basis for groundwater resource development and land subsidence control in Langfang City.

## 1. Introduction

Land subsidence is a slow-onset geological hazard that often leads to deformation or cracking of roads, bridges, and railways. Uneven subsidence can damage both surface and underground structures and infrastructure, while subsidence in coastal areas may exacerbate relative sea level rise, leading to issues such as seawater inundation. This poses a serious threat to economic development and human safety [1–4]. Currently, more than 200 cities and regions worldwide are facing severe threats from land subsidence. In areas such as California (USA), Jakarta (Indonesia), Tokyo (Japan), and Mexico City, land subsidence caused by excessive groundwater extraction has been observed [5–8]. With the rapid development of urbanization and the continuous expansion of population size, groundwater extraction has increased dramatically, leading to increasingly severe land subsidence issues. Regions such as the Yangtze River Delta, the Pearl River Delta, the Yellow River Delta, and the North China Plain have become typical land subsidence areas in China [9–12]. Langfang City is located in the central-eastern part of the North China Plain. Due to the persistent over-extraction of groundwater, groundwater funnels have formed in the urban area of Langfang and some county towns. The subsidence rate in the urban area of Langfang exceeds 60 mm/a, with localized rates reaching up to 80 mm/a. In recent years, with the implementation of water restriction policies and the South-to-North Water Diversion Project, the decline of groundwater levels in Langfang City has significantly slowed, which has played a certain role in controlling the rapid development of land subsidence. The exploitation of groundwater resources and the resulting land subsidence pose a significant challenge to regional economic development. How to balance groundwater extraction with subsidence prevention is an urgent issue that needs to be addressed.

Current research on land subsidence issues primarily focuses on the following aspects: 1) the mechanisms of subsidence formation; 2) subsidence monitoring techniques; 3) the hazards and control of subsidence; 4) numerical simulation and theoretical analysis [13–25]. Numerical simulation analysis plays a crucial role in revealing the mechanisms of land subsidence, predicting subsidence development trends, and optimizing groundwater extraction strategies. In one-dimensional numerical simulations, coupled numerical models are typically established based on groundwater dynamics and one-dimensional consolidation theory. However, such models can only reflect vertical groundwater flow and stratum deformation, and are unable to account for lateral water flow or variations in complex geological formations [13,16,18,21]. To address the limitations of one-dimensional models, researchers have introduced quasi-three-dimensional models. Typically, these models couple the three-dimensional flow of groundwater with the one-dimensional deformation of strata, and they are extensively utilized to analyze the correlation between groundwater flow fields and stratum deformation. A coupled regional land subsidence model, consisting of a three-dimensional groundwater flow model and a one-dimensional viscoelastic-plastic constitutive equation, has been developed to address land subsidence in Shanghai and the Yangtze River Delta region of China. The model was solved using a multi-scale finite element method, and satisfactory results have been achieved [19,23,24]; Meanwhile, researchers obtained land subsidence data for the study area through leveling measurements, GPS surveys, and InSAR measurements. A quasi-three-dimensional land subsidence model was established using Modflow software to predict the evolution trends of land subsidence under different groundwater extraction scenarios [26–33]. Moreover, recent numerical analyses of stratified geomaterials have demonstrated that interlayer heterogeneity and spatial variability can significantly influence the mechanical response and failure mechanisms of geological media [34]. In aquifer–aquitard systems with distinct layered structures, such stratigraphic heterogeneity may likewise affect deformation and subsidence responses, and should therefore be taken into account in investigations of land subsidence induced by groundwater extraction.

However, when the groundwater level declines due to extraction, deformation of the soil skeleton often occurs simultaneously in both vertical and horizontal directions [35–37]. Therefore, it is necessary to consider the three-dimensional deformation of the soil skeleton when simulating land subsidence. Moreover, in the case of land subsidence caused by groundwater extraction, the deformation of the soil skeleton and the variation in pore water pressure are physically coupled. The actual land subsidence problem should be treated as a coupled issue. On the other hand, seepage processes in complex media are characterized by pronounced nonlinearity. Relevant studies have demonstrated that the evolution of the seepage field can significantly influence the deformation and stability of geological media [38]. Therefore, in investigations of stratum deformation induced by groundwater extraction, particular attention should be given to the coupling between the seepage field and the stress field. As the research progressed, researchers developed a three-dimensional fully coupled computational model based on Biot’s consolidation theory to analyze land subsidence issues [39,40]. For multi-causal land subsidence issues, such as those induced by the combined effects of high-rise building loads, groundwater recharge, and coal mining with water extraction, Researchers commonly use commercial numerical simulation software, such as Adina, Flac3D, and Comsol, to conduct fluid-solid coupling analyses, achieving satisfactory results [41,42]. However, in the numerical simulation studies on three-dimensional land subsidence, the soil skeleton of the aquifer system is almost always treated as an elastic material, which fails to reflect the elastic and plastic deformation characteristics of aquitards during groundwater extraction. This study integrates engineering geological and hydrogeological data from Langfang City, employing the Mohr-Coulomb and Cambridge clay constitutive models to characterize the deformation behavior of the aquifers and aquitards’ soil skeletons, respectively. A coupled hydro-mechanical numerical analysis was conducted to investigate the pore water pressure evolution, deformation mechanisms of the aquitards, and the deformation characteristics of the aquifer system under 15 groundwater extraction scenarios. The findings provide a scientific basis for the prevention and control of land subsidence in Langfang City.

## 2. Geological conditions of the study area

### 2.1. Geographic overview

**Fig 1:**
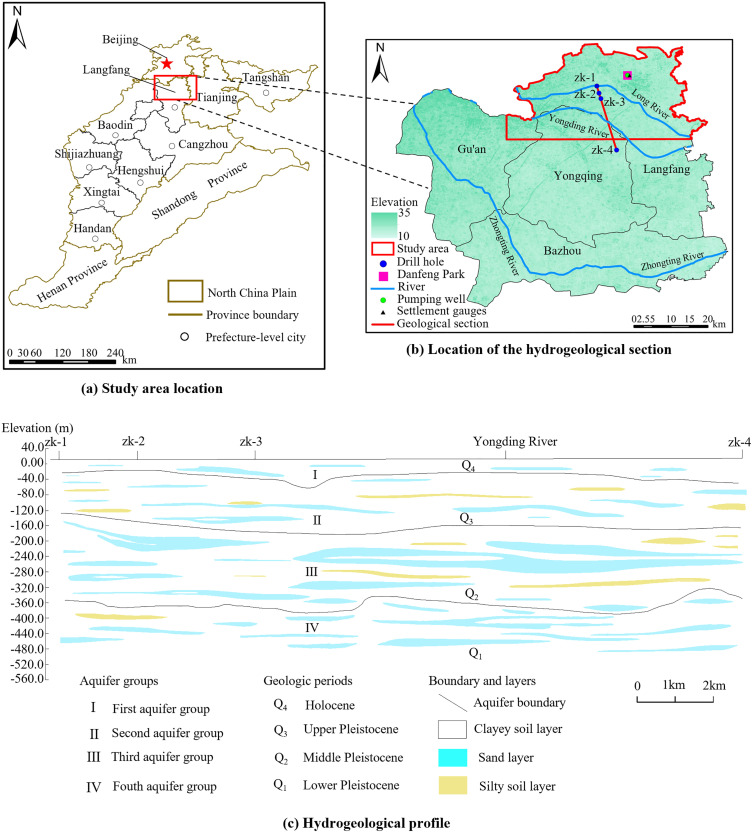
Typical hydrogeological profile and its location in the study area ((a–b) The maps were created by the authors using ArcGIS 10.8.1 (https://www.esri.com/en-us/arcgis/products/arcgis-desktop/resources) based on publicly available Landsat 8 Collection 2 Level-2 data from the Geospatial Data Cloud site, Computer Network Information Center, Chinese Academy of Sciences (http://www.gscloud.cn), (c) Hydrogeological profile of the study area, interpreted and drawn by the authors based on borehole and hydrogeological data).

Langfang City is located in the central-eastern part of the North China Plain (Fig 1a), with a total area of 6,429 km². Its geographical coordinates are 38°28′ ~ 40°15′ E and 116°7′ ~ 117°14′ N. Langfang City has relatively flat topography, predominantly consisting of plains, with an average elevation of approximately 13.0 meters (Fig 1b). Due to the effects of alluvial and fluvial processes, as well as multiple river avulsions, the sediments are interbedded and distributed in a complex manner. Additionally, the influence of wind and human activities has resulted in significant topographical variability, with features such as low hills, depressions, sand dunes, and small alluvial mounds being widely distributed. Langfang City is situated in the mid-latitude zone and experiences a warm temperate continental monsoon climate. The average annual precipitation is 554.9 mm, with uneven seasonal distribution, as precipitation from June to August typically accounts for 70% to 80% of the total annual rainfall. The average annual temperature is 11.9°C, with approximately 2660 hours of sunshine annually. The average annual evaporation is 1,739.2 mm, and the average annual frost-free period is around 183 days. The Feng River, Long River, and Yongding River, which are flood channels, are distributed in the northern, central, and southwestern parts of the city, respectively. All three flow from the northwest to the southeast into the Haihe River, and are part of the Haihe River system.

The study area is located in the northeastern part of Langfang City, as shown in Fig 1b, covering an area of approximately 753.55 km². It includes the central and northern parts of Langfang City, the northern part of Yongqing County, and the northeastern part of Gu’an County. Land subsidence in the urban area of Langfang City began around 1965, and the subsidence area has expanded annually since then. By 2017, the subsidence funnel in the central urban area had reached 138.63 km², with a maximum subsidence exceeding 1.0 m, and the subsidence rate continues at 50 ~ 60 mm per year [43]. Over-extraction of groundwater is the primary cause of land subsidence in this region. Although the government has implemented water restriction policies in recent years, resulting in a slowdown in the subsidence rate, the deformation of deep aquitards continues to develop. As a result, the prevention and control of land subsidence remains a critical challenge.

### 2.2. Hydrogeology

Based on the lithology of the strata, the Quaternary strata in the study area can be divided into 4 aquifer groups (Fig 1c), corresponding to Q_4_, Q_3_, Q_2_, and Q_1_ from top to bottom. The bottom depths of aquifer group I, II, III, and IV are 20 ~ 30m, 130 ~ 150m, 350 ~ 390m, and 510 ~ 520m, respectively. The hydro-chemical types of the aquifer group Ⅰ, Ⅱ, and Ⅳ are primarily HCO_3_-NaMg, while the aquifer group Ⅲ is mainly HCO_3_-NaMg and HCO_3_-CaMg. The mineralization of aquifer group I and II ranges from 1.0 to 2.0 g/L, while the mineralization of aquifer group III and IV is less than 0.5 g/L.

**Fig 2:**
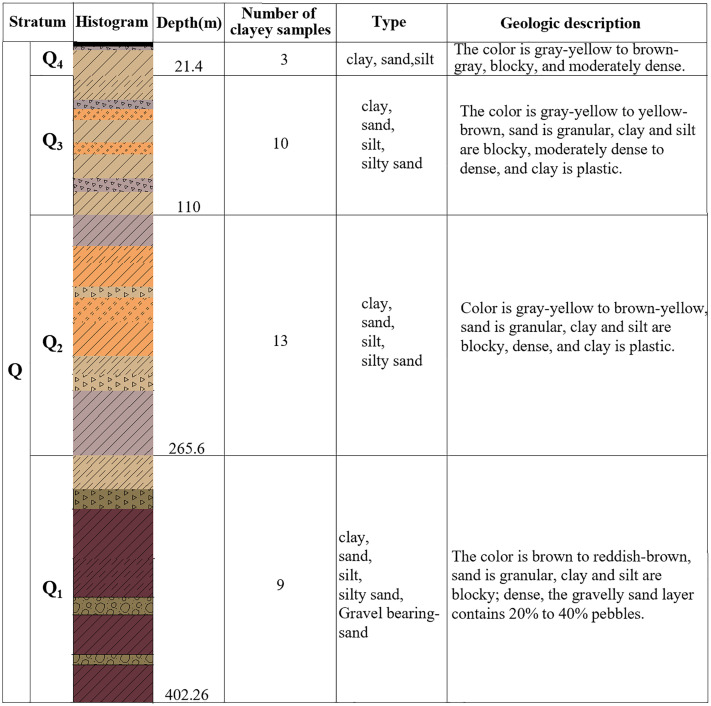
Borehole columnar diagram in the study area.

The Quaternary strata in the study area include the Holocene, Upper, Middle, and Lower Pleistocene. The stratigraphic division is established based on the lithologic column of the zk-2 borehole shown in Fig 1(b) and illustrated in Fig 2. The Holocene (Q_4_) strata are primarily composed of clay, silty clay, and fine sand layers. The Upper Pleistocene (Q_3_) and Middle Pleistocene (Q_2_) strata mainly consist of clayey soil, silty clay, silty sand, and fine sand layers. The Lower Pleistocene strata are primarily composed of clayey soil, silty clay, silty sand, and gravelly sandy soil with pebbles.

**Fig 3:**
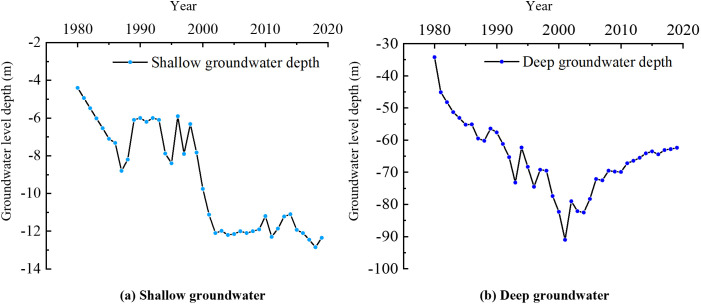
Interannual variation of the average groundwater depth in the study area: (a) shallow groundwater; (b) deep groundwater.

In groundwater studies of the North China Plain, the phreatic water in Aquifer Group I and the semi-confined water in Aquifer Group II are generally classified as shallow groundwater, whereas the water in Aquifer Groups III and IV is classified as deep groundwater. The groundwater depths described in this paper are referenced to the ground surface, which has an elevation of 13.12 m in Langfang City. Over the past 40 years, the shallow groundwater level in Langfang City has generally shown a declining trend, with an average drop of 7.94 m, corresponding to an average annual decline of 0.21 m (Fig 3a). From 1980 to 1986, Langfang City experienced a relatively dry period, during which the extraction of shallow groundwater increased, resulting in a continuous decline in the groundwater level. From 1987 to 1996, it was a relatively wet period, and the groundwater level showed some recovery. From 1997 to 2007, the region entered another dry period, with a significant decline in the groundwater level. After 2008, groundwater extraction decreased, and precipitation increased, entering a relatively stable period. The depth of the groundwater table fluctuated minimally, ranging from −11.0m ~ −13.0m. The changes in the groundwater level were primarily influenced by water resource management efforts and precipitation. From 1980 to 2000, the deep groundwater showed a continuous decline, with the groundwater table depth reaching −34.20 m in 1980 and −91.0 m in 2001. Deep groundwater was the primary source of extraction in Langfang City, and industrial and agricultural water use had an impact on it. After 2000, due to the implementation of the ban on deep groundwater extraction, the groundwater level began to recover and entered a relatively stable period (Fig 3b).

**Fig 4:**
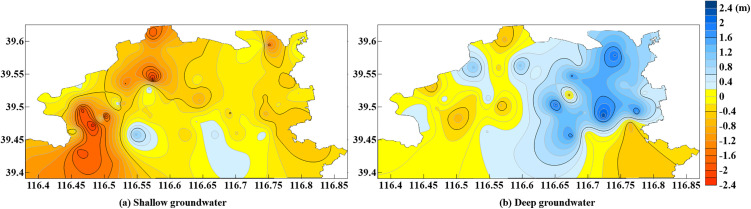
Contour map of groundwater level changes in the study area (June 2018 to July 2019): (a) shallow groundwater; (b) deep groundwater. (The figure was generated by the authors in Golden Software Surfer (https://www.goldensoftware.com/products/surfer/) based on groundwater level change data provided and authorized for use by the China Institute of Geo-Environment Monitoring.).

From June 2018 to July 2019, the China Geological Environment Monitoring Institute conducted groundwater level surveys on 94 wells in the study area. Based on the survey data, a contour map of groundwater level changes was plotted (Fig 4). As shown in the figure, the shallow groundwater level in the study area generally exhibits a downward trend, with a more significant decline in the western region, where the maximum decrease reaches 2.3 m. In the southern region, there is a slight increase in groundwater levels, with the maximum rise being approximately 1.0 m. In other areas, the groundwater level fluctuations are primarily concentrated between −0.5m and 0m, with minimal variation. The deep groundwater level generally exhibits an upward trend, with a more significant rise in the eastern part of the study area, where the maximum increase reaches 2.4m. However, slight declines are observed in the southeastern and western regions.

### 2.3. Compression test of clayey soil

**Fig 5:**
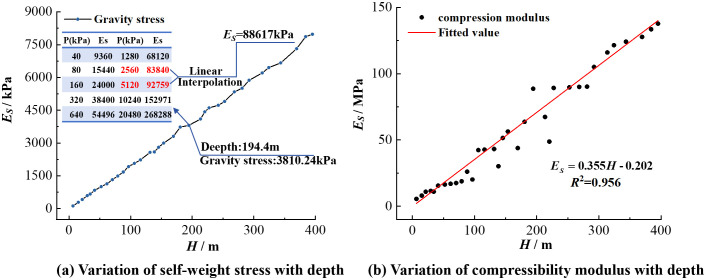
Variation of (a) clay gravity stress and (b) compression modulus with depth.


Basic physical properties and one-dimensional compression consolidation tests were conducted on 35 clayey soil samples (Fig 2) obtained from the zk-2 borehole. Based on the physical property parameters, the gravity stress at different depths can be calculated. For example, at a depth of 194.4 m, the gravity stress is 3810.24 kPa. A stepwise loading scheme was applied: 40kPa → 80kPa → 160kPa → 320 kPa → 640 kPa → 1280kPa → 2650kPa → 5120 kPa → 10240kPa → 20480kPa, and the compression modulus under different loads was measured. By using linear interpolation, the compression modulus corresponding to the gravity stress state was found to be 88,617 kPa (Fig 5a). The relationship between depth and gravity stress was calculated, and subsequently, the relationship between depth and compression modulus (*E*_*S*_) was obtained (Fig 5b). As shown in Fig 5, with increasing depth, gravity stress exhibits a gradual upward trend, and the compression modulus increases with depth, showing a good linear relationship. Using the linear fitting function in Origin software, the experimental data were fitted to obtain the relationship between compression modulus (*E*_*S*_) and gravity stress (*γH*) (Equation 1), where *γ* is the weighted average value of the bulk density of each soil layer at different depths. The coefficient of determination was 0.959, indicating a good fit and accurately reflecting the trend of change between compression modulus and depth.

$$E_{S}=17.79*\gamma H-2695$$ (1)

## 3. Numerical model verification

To systematically analyze the coupled response of groundwater seepage and soil deformation in aquifer – aquitard systems under groundwater extraction, a coupled fluid-solid three-dimensional numerical model is developed in this study. The model framework simultaneously accounts for the interaction between groundwater seepage and the mechanical behavior of the soil skeleton, aiming to characterize the evolution of pore water pressure induced by pumping and its effects on stratum deformation and land subsidence. Considering the differences in structural characteristics and mechanical behavior between aquifers and aquitards, different constitutive relationships are adopted for different media in the model: the Mohr-Coulomb model is used for aquifers, whereas the Cam-Clay model, which can capture yield behavior and plastic deformation under hydrostatic pressure, is employed for aquitards. Based on this framework, the governing equations, model construction, and parameter settings of the numerical model are described in the following sections.

### 3.1. Governing equations of the numerical model

Fully Coupled Seepage-Stress-Deformation Equations [44, 45]:

(1) Equilibrium Equation


$$\nabla \cdot \sigma +{\text{f}}^{b}=0$$ (2)

σ=Ce-αλI (3)

This equation is established based on the assumptions that the pore fluid is incompressible, the soil is saturated, and fluid flow follows Darcy’s law. In the equation, σ is the stress tensor; ${\text{f}}^{b}$ is the body force; C is the stiffness matrix of the soil skeleton constitutive equation; *λ* is the pore water pressure variable; I is the Kronecker delta vector; ∇ is the Laplace operator; e is the strain tensor; and 𝛼 is the Biot-Willis coefficient, representing the contribution of pore water pressure to the effective stress of the porous skeleton; under constant pore pressure conditions, it can also be used to characterize the change in fluid content induced by volumetric strain. Considering that the study object is saturated soil, 𝛼 is often taken as 1.

(2) Continuity equation


$$\frac{\partial \theta }{\partial t}-q^{b}=-\nabla \cdot \text{v}$$ (4)

$$\theta =\alpha e_{v}+\beta \lambda$$ (5)

v=−k·∇λ (6)

In the equation, *θ* represents the fluid content in the soil skeleton, assumed to vary linearly with pore water pressure *λ* and soil skeleton volume strain $e_{v}$; $q^{b}$ is the fluid outflow in the soil skeleton; v is the seepage velocity tensor, which follows Darcy’s law; **k** is the generalized permeability tensor including the effect of fluid viscosity. In this study, the pore fluid is assumed to be water with constant properties, and its viscosity is taken as 1.0 × 10^−3^Pa·s at room temperature; *β* is the proportionality coefficient relating the increment in fluid content to the change in fluid pressure, and represents the rate of fluid- content change induced by fluid-pressure variation under constant volumetric strain, i.e., $\beta ={\left(\frac{\partial \theta }{\partial \lambda }\right)}_{e_{v}=0}$.

It should be noted that Eq. (6) is written in the pressure form adopted in ADINA. Accordingly, **k** corresponds essentially to 𝐾/𝜇, rather than the conventional hydraulic conductivity in units of m/s. Gravity is not neglected in the present formulation; its effect is incorporated through the body-force term in the equilibrium equation and the hydrostatic initial/ reference pressure condition in the coupled seepage-deformation analysis.

Substituting Equations (5) and (6) into Equation (4) gives the continuity equation:


$$\nabla \cdot \left(\text{k}\cdot \nabla \lambda \right)=\alpha \frac{\partial e_{v}}{\partial t}+\beta \frac{\partial \lambda }{\partial t}-q^{b}$$ (7)

(3) Constitutive equation of the soil skeleton [45]

The constitutive relationship of the soil skeleton in the aquifer is modeled using the Mohr-Coulomb elastoplastic model, with the yield function represented as an irregular hexagonal pyramid in the stress space, as shown in Equation (8).


$$f_{\text{MC}}=sin\left(\phi \right)I_{\text{1}}+\frac{\text{1}}{\text{2}}\left(3\left(1-sin\left(\phi \right)\right)sin\left(\delta \right)\right)+\sqrt{\text{3}}\left(3+sin\left(\phi \right)\right)cos\left(\delta \right)\sqrt{J_{\text{2}}}-3ccos\left(\phi \right)$$ (8)

In the equation, *ϕ* is the internal friction angle of the soil; *c* is the cohesion of the soil; *δ* is the Lode angle defined on the deviatoric stress plane; *I*_1_ is the first stress invariant; and *J*_2_ is the second deviatoric stress invariant.

The constitutive relationship of the soil skeleton in the aquitard is modeled using the Cam-clay Model. This model is based on critical state soil mechanics theory and can account for yield behavior under hydrostatic pressure. It is highly suitable for analyzing the plastic deformation in aquitards induced by pumping. The yield function of the Cam-clay Model can be expressed as Equation (9).


$$f_{\text{CC}}=\frac{q^{\text{2}}}{M^{\text{2}}}+P\left(P-P_{\text{0}}\right)$$ (9)

**Fig 6:**
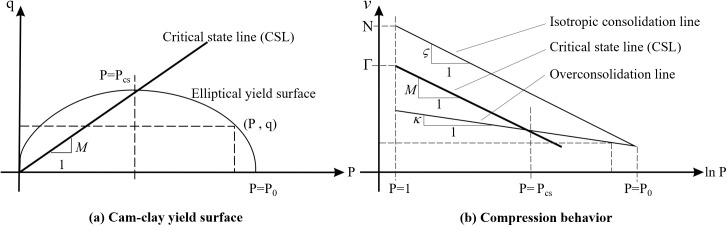
Cam-clay constitutive model [45].

In the equation, *P* is the mean effective stress, $P=\frac{\text{1}}{\text{3}}\left(\sigma_{\text{1}}\text{'}+2\sigma_{\text{3}}\text{'}\right)$ where 𝜎‘ is the effective stress; *q* is the deviatoric stress, cc; *M* is the slope of the critical state line; *P*_0_ is the pre-consolidation pressure, which is the diameter of the ellipsoi; the meaning of each variable is illustrated in Fig 6(a).

The hardening rule is:


$$v=N-\varsigma ln\left(P_{\text{0}}\right)+\kappa \;ln\left(\frac{P_{\text{0}}}{P}\right)$$ (10)

In the equation, *v* is the specific volume; *N* is the specific volume at the isotropic consolidation state; *K* is the effective bulk modulus; *ς*，*κ* and Γ are material constants, where *ς* is the slope of the isotropic consolidation line; *κ* is the slope of the overconsolidation line; the meaning of each variable is illustrated in Fig 6(b) Γ is the specific volume at the critical state and is related to *N*:


Γ=N+(κ−ς)ln P0 (11)

The effective bulk modulus *K* can be expressed as:


$$K=\frac{vP}{\kappa }$$ (12)

The material constant *M* is computed based on *ϕ*. In triaxial compression tests, $M=\frac{6sin\phi }{3-sin\phi }$; in triaxial extension tests, $M=\frac{6sin\phi }{3+sin\phi }$.

During the coupled iterative solution process, due to fluid flow, the pore volume changes. It is assumed that the relationship between the instantaneous porosity ϕ and the initial porosity $\phi^{0}$ is given by Equation (13):


$$\phi =1-\frac{J^{0}}{J}\left(1-\phi 0\right)$$ (13)

The porosity affects the permeability of the soil skeleton, and the relationship between the permeability coefficient and porosity is expressed by Equation (14):


$$\text{k}=\frac{\phi }{\phi^{0}}{\text{k}}^{0}$$ (14)

In the equation, *J* is the Jacobian determinant of the current geometric element; *J*_0_ is the Jacobian determinant of the initial geometric element; and ${\text{k}}^{0}$ is the initial permeability tensor. In this study, the linear update relationship given in the ADINA theory and modeling guide is employed. This relationship can be considered a special case of the general power-law model $\text{k}={\text{k}}^{0}{\left(\phi /\phi^{0}\right)}^{n}$, for 𝑛 = 1.

By simultaneously solving Equations (13) and (14) with Equations (4)-(6) and (2)-(3), and performing finite element discretization of the governing equations, a finite element computational model is established, enabling the couple solution of fluid-stress-deformation in porous media.

### 3.2. The numerical model

**Fig 7:**
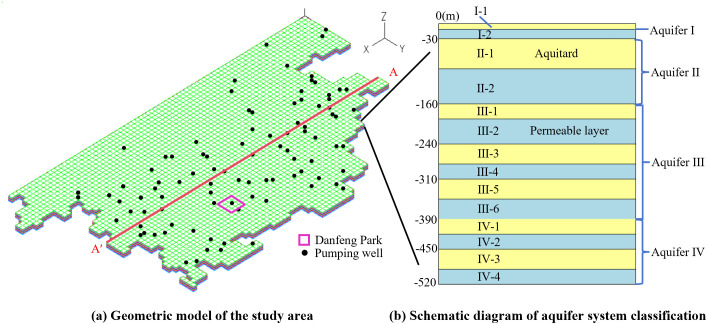
Numerical model of the study area and classification of its aquifer system: **(a)** Geometric model **(b)** Schematic diagram of aquifer system classification.

The established numerical model, including its geometric structure and stratigraphic configuration, is shown in Fig 7. Specifically, Fig 7a illustrates the model domain, mesh discretization, and boundary conditions. The grid was meshed by using the finite element software ADINA, with hexahedral 8-node pore medium elements. The grid was divided in the plane with a length × width of 500m × 500m, resulting in a total of 47,085 model elements and 53,392 nodes (Fig 7a). The selected planar extent covers the main distribution area of pumping wells within the study area, while keeping the model boundaries at an appropriate distance from the primary zone affected by pumping, thus minimizing the influence of artificial boundaries on the simulation results of pore water pressure and stratum deformation. Vertical displacement constraints were applied at the bottom of the model, along with permeable boundaries. Horizontal displacement constraints were applied around the perimeter of the model, and the top of the model was set as a permeable free boundary. These settings are respectively adopted to characterize the support of the deep strata, the lateral confinement, and the hydraulic exchange conditions between the shallow aquifer and the surrounding environment.

Based on the locations of the pumping wells surveyed in 2018 and 2019, 94 pumping wells were placed in the strata (Fig 7a). Prior to applying the pumping load to the model, gravitational field data were imported to eliminate displacements caused by gravity. The vertical structure of the aquifer system in the study area is shown in Fig 7b and can be divided into four aquifer groups from top to bottom. Since aquifer group I is unconfined, this group is set as one aquitard and one aquifer, with the bottom depth at 30.0m. Aquifer group II is slightly confined, with the bottom depth at 160.0m, simulated by setting one aquitard and one aquifer. Aquifer group III is confined, with the bottom depth at 390.0m, simulated by setting three aquitards and aquifers. Aquifer group IV is confined, with the bottom depth at 520.0m, simulated by setting two aquitards and aquifers (Fig 7b).

The relationship between the deformation modulus *E* of the aquitard and the compression modulus *E*_*S*_ of the clayey soil is given in Equation (15).


$$E=\left(1-\frac{2\mu^{\text{2}}}{1-\mu }\right)E_{S}$$ (15)

Using Equations (1) and (15), the deformation modulus corresponding to the top and bottom depths of each aquitard can be calculated. A thickness-weighted averaging method is employed to determine the modulus of an aquitard, as expressed in Equation (16).


$$E_{avg}=\frac{\sum_{i=1}^{n}E_{i}\cdot z_{i}}{\sum_{i=1}^{n}z_{i}}$$ (16)

In the equation: $E_{avg}$ is the weighted average modulus of the aquitard; $E_{i}$ is the modulus of the 𝑖-th clayey soil sample within the layer; $z_{i}$ is the corresponding thickness (m); *n* is the number of clayey soil samples in the layer.

The model parameters of the aquifers obtained through data analysis [44,46–48] are presented in Table 1, while the model parameters of the aquitards are shown in Table 2.

**Table 1:** Model parameters of the aquifers.

Aquifer number	*E* (MPa)	*ν*	*ϕ* (°)	*c* (kPa)	*ρ* (kg/m^3^)	*K*_h_ (m/d)	*K*_v_ (m/d)
Ⅰ-2	145.10	0.25	33	0	1990	6.4	3.2
Ⅱ-2	304.46	0.25	33	0	1980	4.3	2.1
Ⅲ-2	1165.36	0.25	35	0	1940	4.3	2.1
Ⅲ-4	1291.92	0.25	35	0	1950	4.3	2.1
Ⅲ-6	1432.66	0.25	35	0	1920	3.2	1.6
Ⅳ-2	1535.54	0.25	35	0	1870	3.2	1.6
Ⅳ-4	1746.24	0.22	37	0	1870	3.2	1.6

Note: *K*_h_ - Horizontal permeability coefficient; *K*_v_ -Vertical permeability coefficient.

**Table 2:** Model parameters of the aquitards.

aquitard number	*E* (MPa)	*ν*	*c* (kPa)	*ρ* (kg/m^3^)	*OCR*	*K*_h_ (m/d)	*K*_v_ (m/d)	*Pull* (Pa)	*Γ*	*M*	*ς*	*κ*
Ⅰ-1	1.32	0.3	40.97	1920	1	0.008	0.004	10000	1.868	1.05	0.006	0.00155
Ⅱ-1	11.85	0.3	41.91	1980	1	0.008	0.004	10000	1.961	1.07	0.018	0.00300
Ⅲ-1	33.77	0.3	40.56	1990	1	0.004	0.002	10000	1.918	1.24	0.094	0.01174
Ⅲ-3	50.07	0.3	40.03	2050	1	0.004	0.002	10000	1.954	1.22	0.096	0.01216
Ⅲ-5	62.06	0.3	39.95	2040	1	0.0002	0.0001	10000	1.995	1.32	0.098	0.01506
Ⅳ-1	76.65	0.3	40.72	2040	1	0.00008	0.00004	10000	2.310	1.34	0.144	0.01569
Ⅳ-3	86.66	0.3	41.58	2100	1	0.00008	0.00004	10000	2.337	1.37	0.145	0.01613

Note: *Pull* – Initial size of the yield surface.

### 3.3. Verification of the numerical model

This numerical model primarily investigates the variation pattern of pore water pressure during groundwater extraction, and analyzes the deformation characteristics of each aquifer under the influence of pore water pressure, as well as its impact on land subsidence. The main objective is not to precisely predict land subsidence, but rather to study the dynamic variation of pore water pressure under different extraction conditions and its effect on the underground media. The key to model validation lies in examining the deformation response induced by pumping at specific layers. Therefore, data from a monitoring well were selected for validation in order to assess the accuracy and reliability of the numerical model.

Variations in groundwater level and deformation from June 2018 to June 2019 were monitored using the pumping well located in Danfeng Park in the northern part of the study area (longitude 116°40′31.84″, latitude 39°32′51.04″) and the nearby set of layered settlement gauges. The monitoring data were used to validate the reliability of the model calculations, with the water level data sourced from the III aquifer group. The period from June 2018 to June 2019 was designated as the time interval for model validation, with each month considered a separate groundwater extraction phase, and each month treated as one computational time step. Using the measured burial depth data from the monitoring well in June 2018 as the baseline, the data from other months were subtracted from this baseline to obtain the variation of pressure head during the monitoring period, as shown in Table 3. The monitoring data obtained by layered settlement gauges from June 2018 to June 2019 are shown in Table 4.

**Table 3:** Groundwater depth and variation of pressure head in Danfeng Park from June 2018 to June 2019.

Time	Groundwater depth (m)	Variation of pressure head (m)	Time	Groundwater depth (m)	Variation of pressure head (m)
June 2018	77.58	0.00	January 2019	80.21	2.63
July 2018	76.23	−1.35	February 2019	80.87	3.29
August 2018	76.58	−2.36	March 2019	81.11	3.53
September 2018	75.22	−1.00	April 2019	78.46	0.88
October 2018	77.26	−0.32	May 2019	76.92	−0.66
November 2018	79.15	1.57	June 2019	76.28	−1.30
December 2018	79.85	2.27			

**Table 4:** Monitoring data obtained by layered settlement gauges in Danfeng Park from June 2018 to June 2019.

Monitoring borehole number	Depth (m)	Settlement (mm)	Settlement contribution rate (%)
F0-F1	2-67	−2.675	10.38
F1-F2	67-176	0.355	−1.30
F2-F3	176-390	−14.825	57.53
F3-F4	390-520	−8.625	33.46

**Fig 8:**
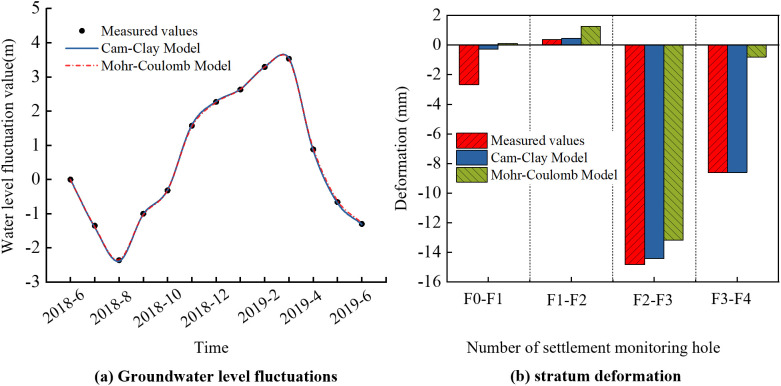
The monitored values versus the model-calculated values of stratum deformation under the variation of pressure head: (a) pressure head (b) stratum deformation.

A localized computational model was established based on the geological conditions near the pumping well in Danfeng Park (Fig 7a). The model measures 2000 m × 2000 m in length and width. It is not shown graphically here due to space limitations. The model parameters and other settings are consistent with those of the larger model shown in Fig 7. To assess the suitability of the constitutive model for the aquitards, two different models were applied: the Mohr-Coulomb model and the Cam-clay Model. Both models used identical values for the deformation modulus *E*, cohesion *c*, and internal friction angle *ϕ*. By adjusting the extraction volume within the model to fit the variation of pressure head, the simulated values were found to align closely with the monitored data, as shown in Fig 8a. The groundwater extraction location in the model is within the aquifers of the aquifer group Ⅲ (Fig 7b). After one year of groundwater extraction, the calculated deformation values at positions F0–F1, F1–F2, F2–F3, and F3–F4 (Table 4) were compared with the monitored values. As shown in Fig 8b, the results from the Mohr-Coulomb model only closely matched the monitored data at the F2–F3 interval, with significant discrepancies at the other positions. In contrast, the deformation values calculated using the Cam-clay Model showed good agreement with the monitoring data across all intervals.

**Fig 9:**
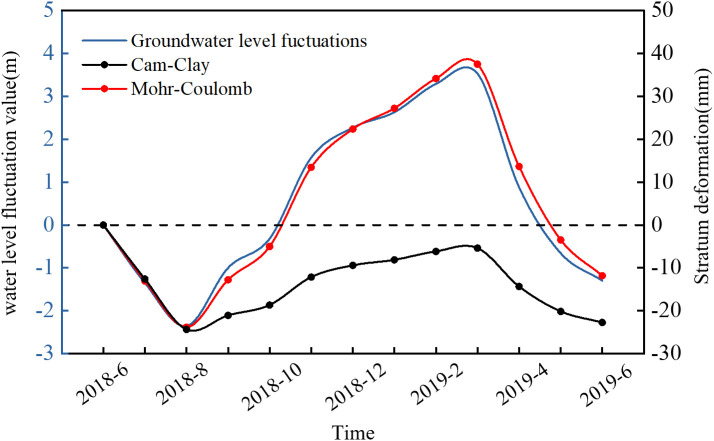
The response of land subsidence to pressure head variation.

The response of land subsidence to the variation of pressure head is shown in Fig 9. Both the Mohr-Coulomb model and the Cam-clay model exhibit a lag effect in the land subsidence with respect to the variation of pressure head. The land subsidence calculated using the Mohr-Coulomb model exhibits a pattern consistent with the variation of pressure head-that is, surface uplift occurs during pressure head increases, and subsidence occurs during pressure head decline. However, this behavior is inconsistent with actual observations, which indicate that land subsidence has continued to progress regardless of the variation of pressure head. The land subsidence calculated using the Cam-clay model remains in a state of subsidence, but the amount of subsidence decreases when the pressure head increases, which is consistent with the observed land subsidence. The primary reason for this phenomenon is that the Mohr-Coulomb model does not capture the yield behavior of soils under hydrostatic pressure. Under the influence of pore water pressure, the soil skeleton remains in an elastic state, resulting in elastic rebound and surface uplift when the pressure head increases. The Cam-clay Model is capable of capturing the yield behavior of soils under hydrostatic pressure. Under a certain level of pore water pressure, the deformation of the soil skeleton becomes irreversible plastic deformation. As a result, no rebound of the ground surface occurs when the pressure head increases.

Overall, when the aquitards are modeled using the Cam-clay Model, the calculated deformation of both the strata and the land subsidence under pressure head aligns more closely with the monitored data. Moreover, this model effectively captures the plastic deformation characteristics of aquitards under pore water pressure, making it a more appropriate choice for such conditions.

The validated numerical model will be used in Section 4 to analyze different groundwater extraction scenarios, and explore the impact of various extraction scenarios on the variations in pore water pressure in the aquifers, deformation of the aquitards, deformation of 4 aquifer groups, and land subsidence.

## 4. Analysis of the effects of land subsidence induced by groundwater extraction

In the numerical model, pumping was conducted simultaneously at 94 wells. The pumping time was set to 12 time steps, with each time step representing one month, corresponding to a continuous pumping duration of one year. In the model, the pumping volume for each well was set to be the same, with a total annual pumping volume of 13,536.0 × 10⁴ m³. Under the condition of unchanged pumping locations, duration, and volume, 15 different groundwater extraction scenarios were implemented. The variations in pore water pressure, deformation of the aquitards, and land subsidence under different extraction scenarios were studied. The 15 extraction scenarios can be divided into three categories: (1) Extraction from only one aquifer group, including four scenarios for aquifer group I, II, III, and IV; (2) Simultaneous extraction from two aquifer groups, including six scenarios for aquifer groups I + II, I + III, I + IV, II + III, II + IV, and III + IV; (3) Simultaneous extraction from multiple aquifer groups, including five scenarios for aquifer groups I + II + III, I + II + IV, I + III + IV, II + III + IV and I + II + III + IV. Specifically, Scenario I refers to pumping from aquifer group I and represents shallow groundwater extraction, whereas Scenario III refers to pumping from aquifer group III and represents deep groundwater extraction. Based on this definition, Scenarios I, III, and I + III are selected as representative cases to compare the land subsidence effects induced by different groundwater extraction strategies.

### 4.1. Characteristics of pore water pressure

**Fig 10:**
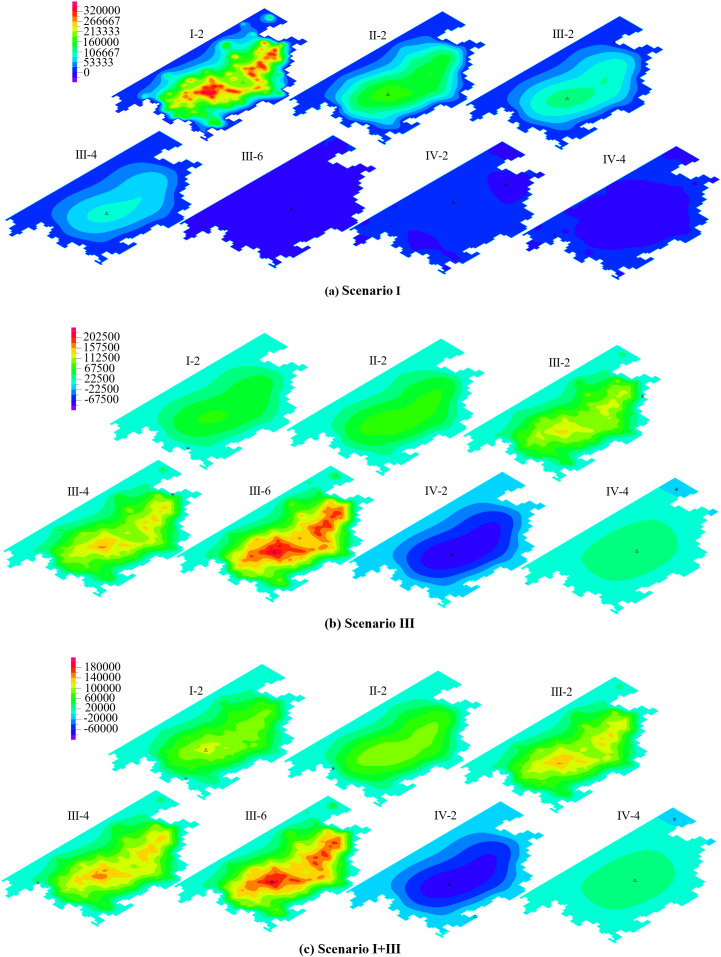
The effect of groundwater exploitation on the pore water pressure in the aquifers.

Groundwater extraction can cause variations in pore water pressure within the strata, which in turn affects the deformation and subsidence of the strata. In a system where tensile stress is positive and compressive stress is negative, the effective stress is expressed as *σ*′ = *σ* + *λ*, where *σ* is the total stress. An increase in pore water pressure leads to a greater effective stress on the soil skeleton units, which in turn causes an increase in compressive deformation and consolidation drainage of the soil skeleton units. When subjected to pumping loads, soil skeleton units at different locations undergo dynamic changes between drainage and water absorption, and their state can be reflected by the variations in positive and negative values of pore water pressure. After one year of continuous pumping, the distribution of pore water pressure in the aquifers shows significant spatial variations (Fig 10). Under the extraction conditions of Scenario I (Fig 10a), the pore water pressure increases significantly in the aquifer I-2 and propagates outward from the pumping center. Due to the low permeability of the aquitards, the transfer of pore water pressure to the deeper aquifers is impeded, resulting in a larger decrease in pore water pressure and a smaller diffusion range. This indicates that shallow groundwater extraction has a limited impact on deeper aquifers. When the pressure reaches aquifer III-6, negative pore water pressure is observed, which is consistent with the findings in the literature [40]. Under the extraction conditions of Scenario III (Fig 10b), the pore water pressure increases significantly in aquifer group III and propagates outward from the pumping center. Vertically, the pore pressure is transferred from aquifer group III upward to aquifer groups I and II, as well as downward to the deeper aquifer group IV. The pore water pressure distribution in Fig 10b shows that deep water extraction has a significant impact on shallow aquifers, but limited impact on deeper aquifers. When the pressure reaches aquifer IV-2, the pore water pressure becomes negative. Under the extraction conditions of Scenario I + III (Fig 10c), the pore water pressure is relatively high in both aquifer group III and aquifer I-2, and it diffuses outward from the pumping center. Vertically, the pressure is transferred downward from aquifer group III, reaching negative values in aquifer IV-2.

**Fig 11:**
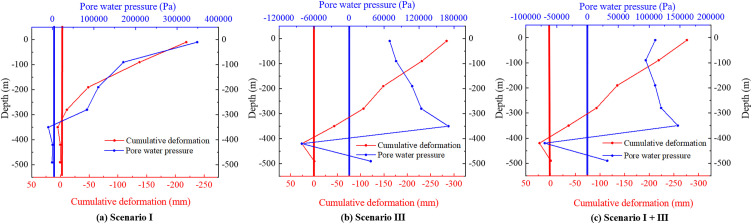
The variation of pore water pressure and cumulative deformation with depth.

The variations of pore water pressure and cumulative deformations with depth are shown in Fig 11. The maximum pore water pressure typically occurs at the pumping location and gradually decreases both upwards and downwards from the pumping point. This phenomenon is attributed to the fact that pumping initially creates a strong hydraulic gradient in the exploited aquifer, and the resulting pore water pressure response subsequently propagates through the aquifers but is significantly retarded when passing through the aquitard because of its low permeability, thus leading to a pronounced layer-by-layer attenuation pattern. During shallow groundwater extraction (Fig 11a), the curves of pore water pressure and cumulative deformation in the aquifer are generally consistent. Locations with higher pore water pressure also show larger cumulative deformation, while at positions where the pore water pressure is negative, the cumulative deformation is positive, indicating localized uplift. This is primarily due to the swelling of the soil skeleton units as they absorb water. During deep groundwater extraction (Fig 11b), the pore water pressure remains highest at the bottom of aquifer group III and gradually decreases both upward and downward. The cumulative deformation increases approximately linearly from the bottom of aquifer group III upward. This is primarily attributed to the fact that pumping from the deep confined aquifer system alters pore water pressure not only within the pumped layer itself, but also triggers a broader redistribution of effective stress across the adjacent overlying and underlying strata, thereby causing both the overlying aquitards and the aquifers to undergo compressive deformation. Negative pore water pressure is observed in aquifer group IV, indicating water absorption and swelling of the soil skeleton. The cumulative deformation at this location is also positive. Under combined upper and lower extraction conditions (Fig 11c), the deformation and pore water pressure of the aquifers are more similar to those observed under Scenario III. Deep groundwater extraction has a more significant impact on cumulative deformation. Overall, due to the influence of aquitards during pumping, negative pore water pressure is observed throughout the aquifers, accompanied by localized minor uplift. The low permeability of the aquitard alters the hydraulic connectivity between strata, resulting in non-uniform attenuation of pore water pressure during vertical transmission and consequently leading to differentiated responses of soil skeleton units at different stratigraphic levels, manifested as drainage-induced compression or water-uptake-induced expansion.

### 4.2. Deformation characteristics of aquitards

#### 4.2.1. Deformation behavior.

**Fig 12:**
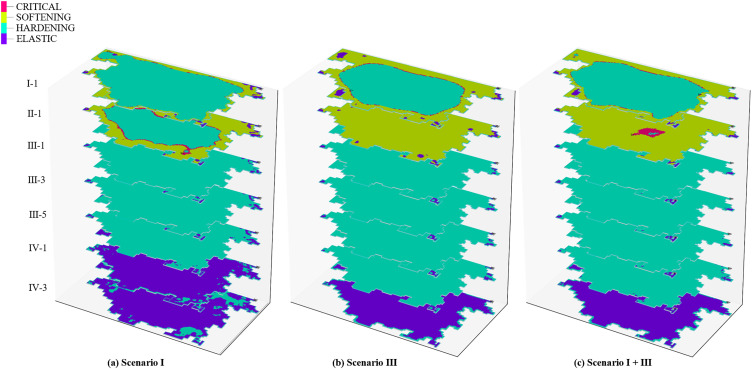
The effect of groundwater exploitation on the deformation behavior of aquitards.

Aquitards are primarily composed of fine-grained soils such as clayey and silty soils, and exhibit certain compressibility. Under extraction conditions, the compression of aquitards is a significant driving factor for land subsidence. In multi-layered groundwater systems, their cumulative deformation can lead to substantial subsidence [49]. During groundwater extraction, the stress state of aquitards changes. As the pumping time increases, the additional stress on the aquitards exceeds their yield stress, causing the aquitards to transition from elastic deformation to irreversible plastic deformation. After one year of groundwater extraction, the deformation of the aquitards is shown in Fig 12. Almost all of the aquitards within the pumping influence zone have undergone plastic deformation. Under the extraction conditions of Scenario I (Fig 12a), strain hardening occurred in most of the aquitard I-1, while a small portion around the edges exhibited strain softening plastic deformation. In the aquitard II-1, strain hardening occurred in the central region, while strain softening was observed around the periphery. The aquitards in aquifer group III exhibited overall strain hardening plastic deformation, and the lower aquitard IV-1 displayed elastic deformation. This is consistent with the locations of negative pore water pressure observed in Fig 10a, which represent the depth of influence of shallow groundwater extraction on the deeper strata. Under the extraction conditions of Scenario III (Fig 12b), plastic deformation in the aquitard I-1 showed strain hardening in the central region and strain softening around the edges. In the aquitard II-1, most areas experienced strain softening, while a small portion remained in elastic deformation. The deeper strata mainly exhibited strain hardening, and the depth of plastic deformation extended further than under Scenario I. Under the extraction conditions of Scenario I + III, the deformation is primarily influenced by the extraction from aquifer group III, and its deformation characteristics are similar to the results under Scenario III (Fig 12c). The difference is that the aquitard II-1 reaches a critical state.

The reasons for strain hardening, softening, and the critical state in aquitards are as follows: during the pumping process, significant changes occur in the pore structure of the aquitards. The soil volume compresses, and the particles become more tightly arranged, resulting in an increase in the initial strength of the soil, thereby leading to strain hardening. However, once plastic deformation reaches a certain level, the soil skeleton slides, distorts, or rotates. This reduces inter-particle contact and frictional forces, causing strain softening. In the Cam-clay Model, strain hardening is primarily influenced by the model parameters *ς* and *M*. *ς* represents the compressibility of the soil in the normally consolidated state, reflecting the sensitivity of the soil’s pore structure to changes in effective stress. *M* is the slope of the critical state line and is closely related to the soil’s internal friction characteristics and critical state strength. The differences in parameters across different aquifer groups are mainly attributed to variations in burial depth, initial effective stress levels, and long-term consolidation history. Below the aquifer group II, the aquitards are at greater burial depths and have been subjected to higher overburden pressures for extended periods. This results in higher consolidation and a relatively denser structure, leading to larger values of *ς* and *M* (Table 2). Consequently, the plastic deformation in these layers exhibits strain hardening, as shown in Fig 12. Due to the shallower burial depths and lower initial effective stress levels of aquifer groups I and II, the soil structure is relatively loose, resulting in smaller values of *ς* and *M*. As a result, the plastic deformation in these two aquifer groups exhibits strain softening, as shown in Fig 12. Under extraction conditions of Scenario I + III, a local critical zone developed in aquitard II-1 (Fig 12c), indicating that the stress state in this region had reached the critical state line defined by the Cam-clay model and had entered a regime of stable plastic flow. At this stage, the soil could still continue to deform plastically, but no longer showed pronounced strain hardening or strain softening, while the rate of volumetric compression gradually decreased. This phenomenon suggests that, under the combined pumping of the upper and lower aquifer systems, the aquitard in Aquifer Group II is simultaneously influenced by pore water pressure variations in the overlying and underlying layers, thereby rendering local areas more prone to reaching the critical state.

#### 4.2.2. Contribution of aquitard deformation to land subsidence.

The deformation of aquitards mainly depends on the thickness of the strata, the compression modulus, variations in groundwater levels, and the characteristics of plastic deformation. The aquitards in the four aquifer groups in this area consist of seven layers (Table 2), with a total thickness of 250.0 m. The thickness and deformation of the aquitards are shown in Table 5. Under the extraction conditions of Scenario I, the total deformation of the aquitards is −171.76 mm, accounting for only 68.25% of the total deformation of the entire aquifer group. This is mainly due to the extraction location being in the upper phreatic layer, which has a relatively high horizontal permeability coefficient, leading to significant water supply on both sides. The reduction in pore water pressure is concentrated mainly in the vicinity of the pumped layer and progressively attenuates during downward transmission because of the low-permeability barrier effect of the aquitard. Consequently, the increment in effective stress within the deeper aquitards is relatively small, resulting in limited consolidation compression. Under the extraction conditions of Scenario III, the total deformation of the aquitards reaches −257.84 mm, accounting for 87.93% of the total deformation. This is mainly because pumping from the deep confined aquifer system causes a widespread decline in pore water pressure, thereby increasing the effective stress in the overlying and adjacent aquitards. Owing to their low permeability and poor drainage conditions, the deeper aquitards are more prone to delayed consolidation, and some of them enter a stage of plastic compression under continuously increasing effective stress, resulting in substantial irreversible deformation. Under the extraction conditions of Scenario I + III, the total deformation of the aquitards falls between the values observed in the previous two scenarios. This is primarily due to the extraction of both the shallow phreatic layer and the deep confined groundwater, while maintaining a constant pumping volume.

**Table 5:** The thickness and deformation of the aquitards.

Extraction Scenario	Number	Thickness (m)	Deformation of each layer (mm)	Total deformation (/mm)	Deformation ratio of each layer	Total deformation ratio
Scenario I	Ⅰ-1	10	−29.899	−171.76	11.880%	68.25%
	Ⅱ-1	60	−34.804		13.829%	
	Ⅲ-1	30	−57.475		22.838%	
	Ⅲ-3	40	−36.639		14.558%	
	Ⅲ-5	40	−15.134		6.013%	
	Ⅳ-1	30	2.908		−1.156%	
	Ⅳ-3	40	−0.718		0.285%	
Scenario III	Ⅰ-1	10	−4.250	−257.84	1.449%	87.93%
	Ⅱ-1	60	−8.039		2.742%	
	Ⅲ-1	30	−42.492		14.491%	
	Ⅲ-3	40	−53.917		18.387%	
	Ⅲ-5	40	−79.167		26.998%	
	Ⅳ-1	30	−95.533		32.579%	
	Ⅳ-3	40	25.563		−8.718%	
Scenario I + III	Ⅰ-1	10	−8.882	−247.65	3.067%	85.52%
	Ⅱ-1	60	−11.632		4.017%	
	Ⅲ-1	30	−44.933		15.516%	
	Ⅲ-3	40	−51.868		17.910%	
	Ⅲ-5	40	−70.429		24.319%	
	Ⅳ-1	30	−81.820		28.253%	
	Ⅳ-3	40	21.910		−7.565%	

#### 4.2.3. Differences in deformation characteristics between aquitards and aquifers.

**Fig 13:**
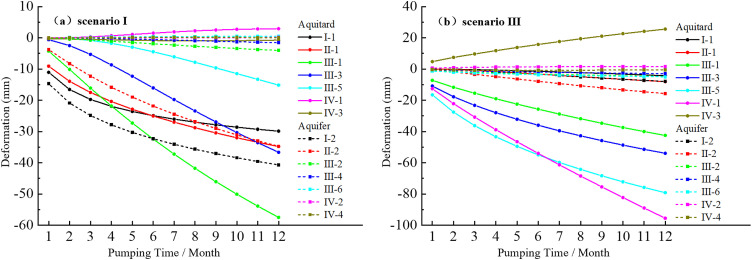
Deformation of aquitards and aquifers under scenarios of shallow and deep groundwater extraction.

Under both shallow and deep groundwater extraction conditions, the deformation of each aquifer and aquitard varies with pumping time, as shown in Fig 13. Under the pumping conditions of Scenario I (shallow layer), the deformation of the aquitard in the deep aquifer group III increases rapidly. As the pumping time progresses, the curve shows a gradual upward trend. However, the deformation of the shallow strata increases rapidly during the first 4 months of pumping, after which the deformation curve gradually flattens as pumping time increases. This is primarily due to the extraction location being in the shallow layers, where pore water pressure increases rapidly in the short term. As the pumping-recharge equilibrium is reached, the increase in pore water pressure stabilizes. In the deeper aquitard IV-1, localized uplift occurs after 4 months of pumping, while the deformation in other deeper strata remains relatively small. Under the pumping conditions of Scenario III (deep layer), the deformation curves of the aquitards III-1, III-3, III-5 and IV-1 show a progressively increasing trend with pumping time. The deformation in these aquitards is relatively large. However, the deformation in the shallow strata remains relatively small during the pumping period, primarily due to the extraction location being in the deep layers, which limits the upward transmission of pore water pressure. In the deeper IV-3 aquitard, localized uplift occurs immediately after pumping begins. As the pumping time increases, the uplift gradually increases, primarily due to the negative pore water pressure at this location, which causes the soil skeleton units to absorb water.

**Fig 14:**
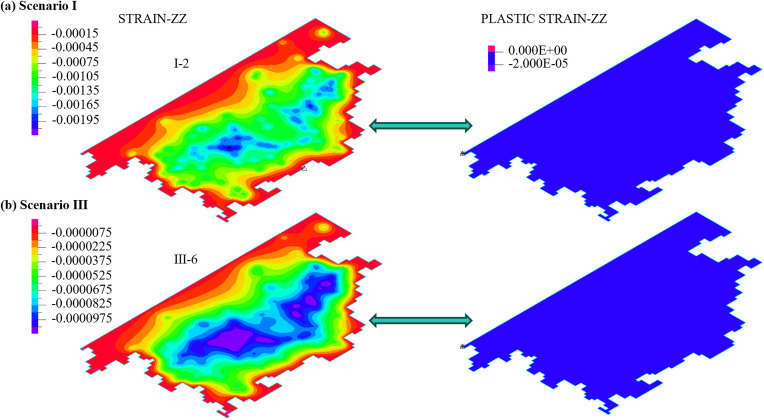
The strain and plastic strain in the Z-direction of the aquifer under shallow and deep excavation conditions.

Under the extraction conditions of Scenario I (shallow layer), the layer with the highest pore water pressure is the aquifer I-2 (Fig 10a). Under the extraction conditions of Scenario III (deep layer), the layer with the highest pore water pressure is the aquifer III-6 (Fig 10b). The Z-direction strain and plastic strain of these two aquifers are shown in Fig 14. The maximum Z-direction strain is concentrated near the pumping wells, with relatively small magnitudes (Fig 14a), indicating that under high pore water pressure conditions, the strain in the aquifers remains small. However, despite the maximum pore water pressure occurring in the aquifers, their plastic strain remains zero (Fig 14b), indicating that the deformation in these layers is entirely elastic in nature. This is mainly because the Mohr-Coulomb model does not capture yield behavior under hydrostatic pressure conditions. Previous studies [44,46–48,50,51] have employed the Mohr-Coulomb model to describe both aquifers and aquitards, which in practice reflects only their elastic deformation behavior. Theoretically, this model is incapable of capturing the plastic deformation characteristics of aquitards.

### 4.3. Deformation characteristics of aquifer groups

**Fig 15:**
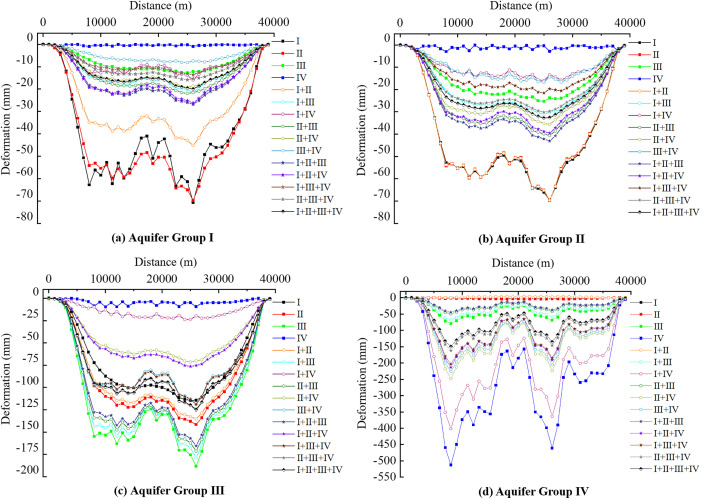
Deformation curves of 4 aquifer groups along Profile A-A′ under different exploitation scenarios.

The strata in the study area primarily consist of four aquifer groups. The deformation values at the nodes of each aquifer group unit along the extraction profile A-A’ (Fig 7a) were obtained, and the deformation variations of the four aquifer groups along profile A–A’ are presented in Fig 15. Under the 15 groundwater extraction scenarios, when the extraction location is within a specific aquifer group, the extraction has the greatest impact on the deformation of that aquifer group. The next most influential scenarios are those that include the extraction location in combination with other aquifer groups, such as Scenario I + II and Scenario II. Scenarios with minimal impact on aquifer deformation are those where the extraction location is farther from the aquifer group, such as Scenario IV and Scenario III + IV (Fig 15a). Under the 15 extraction scenarios, the maximum deformations of aquifer groups I and II are 70.59 mm and 69.64 mm, respectively (Figs 15a and 15b); while the maximum deformations of aquifer groups III and IV reach 188.31 mm and 512.65 mm, respectively (Figs 15c and 15d). This indicates that, given a specific extraction volume, pumping leads to smaller deformations in the shallow aquifer groups and larger deformations in the deeper aquifer groups.

### 4.4. Characteristics of land subsidence under various groundwater extraction scenarios

Under multi-aquifer extraction conditions, the hydraulic connection between aquifers exhibits nonlinear characteristics. Differences in the permeability and recharge of aquitards result in local aquifer water level decline or enhanced hydraulic connection. Additionally, the uneven deformation of the strata makes it difficult to accurately determine the pattern of land subsidence. However, through numerical modeling analysis conducted in this region, certain characteristics of land subsidence can be obtained.

**Fig 16:**
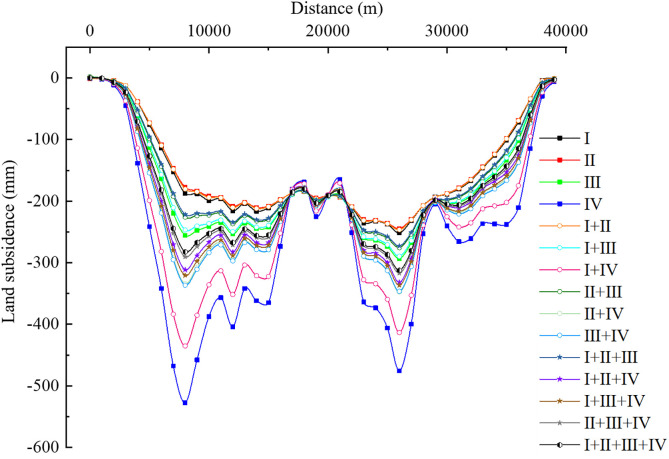
The variation curves of land subsidence along profile A-A′ under different exploitation scenarios.

Under the 15 groundwater extraction scenarios, the variation of land subsidence along profile A-A’ is shown in Fig 16. Overall, the deeper the aquifer extracted, the greater the land subsidence, i.e., Scenario IV > Scenario III > Scenario II > Scenario I under the same pumping volume. Combination scenarios that include Scenario IV result in relatively large land subsidence. This indicates that, under the same total pumping volume, deep groundwater extraction is more likely to induce significant land subsidence, mainly because deep pumping causes a more extensive decline in pore water pressure and a broader redistribution of effective stress, thereby promoting the consolidation compression of aquitards and ultimately resulting in greater cumulative subsidence. In multi-aquifer systems, land subsidence is primarily caused by the drainage consolidation of compressible aquitards, and the magnitude of subsidence does not exhibit a simple monotonic relationship with aquifer depth. When the aquitards in deep aquifer groups are relatively thick and highly compressible, pumping can induce delayed consolidation, resulting in sustained and pronounced subsidence. Numerical simulation studies show that, over the long term, deep groundwater extraction can cause subsidence comparable to, or even greater than, that from shallow extraction. Studies by Chen et al. and Yang et al. also indicate that deep aquifer groups contribute significantly to land subsidence [52,53]. If the minimum land subsidence is considered the control criterion for groundwater extraction, then it is advisable to select either the individual extraction of aquifer groups I or II, or shallow combination extraction scenarios (e.g., Scenario I + II). Extraction of deeper aquifers typically leads to larger subsidence and affects a wide area, thus it is recommended to avoid the solo or large-scale extraction of deep groundwater. In reality, aquifer groups I and II in this area are greatly influenced by atmospheric precipitation, have poor water quality, and are subject to limited extraction. The main extraction layers are aquifer groups III and IV. As seen in Fig 16, the land subsidence caused by the individual extraction of aquifer groups III and IV is significant. From the perspective of balancing the extraction of high-quality groundwater while avoiding large land subsidence, it is recommended to adopt the extraction scenario of I + II + III.

## 5. Conclusions

Using coupled seepage-stress-deformation numerical simulation techniques, this study analyzed the pore water pressure, deformation characteristics of aquitards and aquifer groups under 15 groundwater extraction scenarios in Langfang City, as well as the influence of extraction scenarios on land subsidence. The main conclusions are as follows:

(1) The Quaternary strata in Langfang City primarily consist of four aquifer groups. Based on the lithology and thickness of the strata, the aquifer groups are divided into seven aquifers and seven aquitards. Based on groundwater level and monitoring data by layered settlement gauges from Danfeng Park, the model was calibrated and validated. The results show that the deformation values calculated using the Mohr-Coulomb model for aquitards deviate significantly from the monitoring data, and the deformation increases with the rise in groundwater levels, which contradicts the actual observation of irreversible land subsidence. The reason for this phenomenon is that the Mohr-Coulomb model cannot capture the yield characteristics under hydrostatic pressure. Under the influence of water pressure, the soil skeleton deformation remains elastic, leading to rebound when the water level rises. The Cam-clay model can capture yield characteristics under hydrostatic pressure. Under the influence of water pressure, the soil skeleton undergoes irreversible plastic deformation, making it more suitable for describing aquitards.
(2) Under the pumping load, the distribution of pore water pressure in different aquifers shows significant spatial variation. Shallow pumping mainly affects the shallow aquifer, and the downward propagation of pore-water pressure is significantly impeded by the low permeability of the aquitards. In contrast, deep pumping not only causes a greater decline in pore-water pressure within the pumped layer itself, but also exerts a stronger disturbance on the overlying aquifer system. The occurrence of negative pore-water pressure and slight uplift in local strata reflects the water-absorption-induced swelling response of soil skeleton units under conditions of variable interlayer hydraulic connectivity.
(3) Within the influence range of groundwater extraction, the deformation of aquitards is primarily plastic, including strain hardening, strain softening, and plastic deformation at the critical state. In the Cam-clay model, strain hardening is primarily influenced by the model parameters *ς* and *M.* In the strata below aquifer group II, the relatively large values of these two parameters lead to strain-hardening plastic deformation; strain softening is primarily controlled by the model parameter *M*. Since the parameter *M* is relatively small in the strata above aquifer group II, their plastic deformation exhibits strain softening. As strain softening or hardening progresses, when the stress and porosity reach the critical state conditions, the stress-strain curve of the aquitards levels off, indicating that it has reached the critical state. Under the same extraction volume, shallow groundwater extraction results in less plastic deformation in aquitards and contributes less to land subsidence compared to deep groundwater extraction.
(4) Among the 15 groundwater extraction scenarios, when the extraction site is located within a specific aquifer group, the pumping has the greatest impact on the deformation of that aquifer group. Under a constant extraction volume, the magnitude and extent of land subsidence increase with the depth of the extraction layer, this indicates that deep groundwater extraction is more likely to induce subsidence over a larger area and with a stronger cumulative effect. From the perspective of controlling land subsidence, it is recommended to select either the individual extraction of aquifer groups I or II, or a shallow combination extraction scenario. If both the development of high-quality groundwater resources and the control of land subsidence are considered, it is advisable to prioritize the extraction scenario of I + II + III. These findings can provide a theoretical basis and engineering guidance for optimizing groundwater exploitation and mitigating land subsidence in Langfang City.
